# Prenatal magnetic resonance imaging findings of tuberous sclerosis complex in fetuses

**DOI:** 10.3389/fonc.2026.1846988

**Published:** 2026-07-08

**Authors:** Linjun Xie, Yingkun Guo, Wei Bai

**Affiliations:** 1 Department of Radiology, Medical Imaging Center, West China Second University Hospital, Sichuan University, Chengdu, Sichuan, China; 2 Key Laboratory of Birth Defects and Related Diseases of Women and Children (Sichuan University), Ministry of Education; Development and Related Diseases of Women and Children Key Laboratory of Sichuan Province; Children’s Medicine Key Laboratory of Sichuan Province, West China Second University Hospital, Sichuan University, Chengdu, Sichuan, China

**Keywords:** cardiac rhabdomyoma, cortical dysplasia, fetal MRI, prenatal diagnosis, tuberous sclerosis complex

## Abstract

**Objective:**

This study aimed to characterize the prenatal imaging features of brain and cardiac lesions associated with tuberous sclerosis complex (TSC), with particular focus on fetal magnetic resonance imaging (MRI) findings, and to explore the complementary role of fetal MRI, fetal echocardiography, and genetic testing in prenatal evaluation.

**Method:**

This retrospective study analyzed prenatal neurocardiac MRI findings in fetuses diagnosed with TSC or cardiac rhabdomyomas through genetic and/or clinical assessment between 2018 and 2025.

**Results:**

Twenty-four fetuses with TSC were included, 23 underwent fetal brain MRI, which revealed abnormalities in 15 cases, including subependymal nodules, cortical dysplasias, and subependymal giant cell astrocytoma. Subependymal nodules appear as hypointense on T2-weighted imaging (T2WI) (14/14, 100%), and the vast majority appear as hyperintense on T1-weighted imaging (T1WI) (12/14, 85.7%), while most show hyperintensity on diffusion-weighted imaging (DWI) (11/14, 78.6%). Cortical dysplasia included cortical tubers and white matter migrational abnormalities. Cortical dysplasia shows hyperintensity on T1WI in all cases (8/8, 100%), hypointensity on T2WI (6/8, 75%), and predominantly hyperintensity on DWI (6/8, 75%). Subependymal giant cell astrocytoma appears hypointense on T2WI and hyperintense on T1WI. According to our research, T1WI was superior to T2WI in demonstrating cortical dysplasia. Intracranial lesions in tuberous sclerosis complex manifest as hyperintense on T1WI (contrasting with the dark background of white matter), a phenomenon often referred to as the “bright spot” sign. Cardiac MRI was performed in 12 of the 24 fetuses. Cardiac rhabdomyomas appear as hyperintense signals on T2WI against the dark background of the myocardium (12/12, 100%), a finding commonly referred to as the “bright spot” sign.

**Conclusion:**

The “bright spot” sign on MRI has significant diagnostic value for fetal TSC, particularly for identifying cortical dysplasia and cardiac rhabdomyoma. In cases of suspected TSC or cardiac rhabdomyoma, combining prenatal ultrasound with MRI enhances diagnostic accuracy, facilitating better clinical management.

## Introduction

Tuberous sclerosis complex (TSC) is a rare autosomal dominant genetic disorder that may cause multisystem tumors, most commonly involving the skin, brain, heart, and kidneys (1). The reported incidence of TSC is 1 in 6,000 live births (2). Approximately 35% of cases exhibit familial inheritance, whereas 65% occur sporadically (3–6). Central nervous system (CNS) involvement occurs in most patients, frequently presenting as refractory epilepsy and varying degrees of intellectual disability. These manifestations often lead to poor neurodevelopmental outcomes, emphasizing the need for accurate prenatal detection (7, 8). The prognosis of cardiac rhabdomyoma primarily depends on tumor size, location, clinical symptoms, and association with TSC. Severe complications, such as heart failure and arrhythmias, may occur, necessitating thorough evaluation to guide perinatal management. Early prenatal screening and diagnosis are crucial for developing appropriate clinical and interventional strategies. Ultrasound remains the first-line imaging method for prenatal screening and diagnosis (9). However, with advances in rapid magnetic resonance imaging (MRI) techniques, fetal MRI has become an essential complementary tool, providing additional diagnostic information beyond ultrasound (10). Compared with ultrasound, fetal brain MRI offers superior visualization of intracranial abnormalities. Some studies have explored its role in the prenatal diagnosis of TSC (11, 12). Even fewer have examined the prenatal cardiac MRI features of TSC. Therefore, this study aims to describe the prenatal brain and cardiac MRI findings in fetuses with TSC and review the relevant literature.

## Materials and methods

This study retrospectively analyzed prenatal brain and cardiac MRI data from fetuses diagnosed with TSC through genetic and/or clinical assessment at our institution between May 2018 and August 2025. All patients underwent systematic prenatal ultrasound and targeted MRI. This study was approved by the Institutional Review Board/Medical Ethics Committee of our hospital.

### Prenatal ultrasound

Following the International Society of Ultrasound in Obstetrics and Gynecology (ISUOG) guidelines, two experienced fetal ultrasound specialists performed comprehensive fetal systemic structural ultrasound scans during the second trimester using a 2–9-MHz curved array transducer (GE E8, Philips EPIQ 7 color Doppler ultrasound diagnostic systems). Examinations were conducted at 20–24 weeks of gestation. When brain or cardiac abnormalities were detected, lesion location, size, and echogenicity were documented in detail, and patients were referred for targeted follow-up. Furthermore, other fetal systems were thoroughly examined for structural anomalies.

### Prenatal MRI

Pregnant women were scanned mainly in the supine feet-first position, with lateral positioning as needed. No sedation was administered. MRI protocols adhered to the Chinese expert consensus on fetal MRI, ensuring that specific absorption rate (SAR) remained within recommended limits. Imaging was performed on a 1.5T MR scanner (Achieva dStream, Philips Healthcare, Best, Netherlands) with a 16-channel body phased-array coil.

Fetal brain MRI acquired axial, coronal, and sagittal images using sequences including 3D volume interpolated rapid T1-weighted imaging (T1WI) sequence (slice thickness, 4–5 mm; TR/TE, 4.5 ms/2.1 ms; flip angle, 8°), T2-weighted (T2WI) single-shot turbo spin-echo sequence (slice thickness, 4–5 mm; repetition time/echo time (TR/TE), 1300 ms/100 ms; flip angle, 90°), balanced turbo field echo (BTFE) (slice thickness, 4–5 mm; TR/TE, 5.3 ms/2.7 ms; flip angle, 70°), and diffusion-weighted imaging (DWI) with b-values of 0 and 800 s/mm².

Fetal cardiac MRI included transverse, coronal, and sagittal images from the thoracic inlet to below the diaphragm. Sequences comprised T1WI (slice thickness, 4–6 mm; TR/TE, 4.2 ms/2.0 ms; flip angle, 8°), T2WI (slice thickness, 4–6 mm; TR/TE, 1200 ms/120 ms; flip angle, 90°), BTFE (slice thickness, 4–6 mm; TR/TE, 4.8 ms/2.4 ms; flip angle, 70°), and DWI with b-values of 0 and 800 s/mm².

### Statistical analysis

Two prenatal diagnostic physicians reviewed fetuses with abnormal MRI findings to confirm diagnoses. Discrepancies were resolved through consensus with at least three physicians. Primary observations included lesion location, size, quantity, and signal characteristics, which are summarized and analyzed. Correlations between the number of cardiac and brain lesions, as well as between genetic findings and lesion counts, were assessed. Participants were divided into two groups based on gestational age (>26 weeks or not), and lesion prevalence was compared. A subgroup analysis was conducted based on the diagnostic modality, namely genetic confirmation versus imaging-based diagnosis. Categorical variables are expressed as frequencies (percentages) and were compared using Pearson’s χ2 test or Fisher’s exact test. Statistical analyses were performed using GraphPad Prism (version 7.0; GraphPad Software, San Diego, CA, USA). A two-tailed *p-*value < 0.05 was considered statistically significant.

## Results

**Figure 1:**
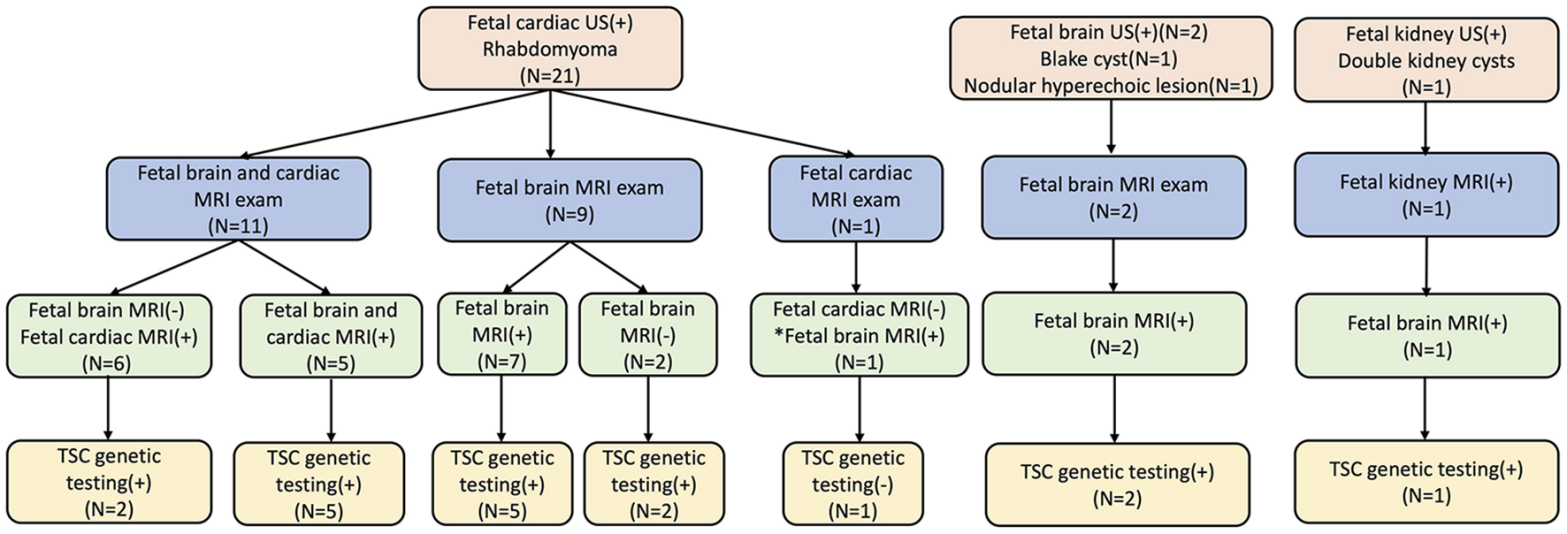
Flow chart for confirming the diagnosis of tuberous sclerosis complex. *MRI localizer images revealed brain lesions. MRI, magnetic resonance imaging; US, ultrasound.

The 24 pregnant women ranged in age from 25 to 40 years (mean, 30.83 ± 3.92 years) and in gestational age from 21 to 38 weeks (mean, 28.56 ± 4.77 weeks). Two pregnancies were twin gestations. The clinical characteristics are summarized in **Table 1**. **Figure 1** depicts the overall flowchart of this study.

**Table 1:** Clinical information and follow-up results of 24 fetuses with prenatal diagnosis of TSC.

No	Diagnosis	Mutation	Parental mutation	GA (weeks)	Maternal age (years)	Brain	Cardiac	Other organ involvement	Pregnancy outcome
1	Clinical	/	/	32+2	35	Positive	Positive	Kidney	Lost to follow-up
2	Clinical	/	/	34+1	29	Positive	Positive	Negative	Pregnancy termination
3	Clinical	/	/	38+1	30	Positive	Positive	Negative	Pregnancy termination
4	Genetic	TSC1	Father	38+1	40	Negative	Positive	Negative	Pregnancy termination
5	Genetic	TSC2	Mother	24+2	37	Positive	Negative	Negative	Pregnancy termination
6	Clinical	/	/	26	29	Positive	Positive	Negative	Pregnancy termination
7	Clinical	/	/	29+1	30	Positive	Positive	Negative	Lost to follow-up
8	Clinical	/	/	25+5	25	Positive*	Positive	Negative	Pregnancy termination
9	Genetic	TSC2	Father	24+3	27	Negative	Positive	Negative	Term birth
10	Genetic	TSC2	Negative	31	29	Positive	Negative	Kidney	Pregnancy termination
11#	Genetic	TSC2	/	25+2	34	Negative	Positive	Negative	Fetal reduction
12	Genetic	TSC2	Father	34+2	32	Positive	Positive	Negative	Pregnancy termination
13	Genetic	TSC2	/	32+1	25	Positive	Positive	Negative	Pregnancy termination
14	Genetic	TSC2	/	23+2	26	Positive	Positive	Negative	Lost to follow-up
15	Genetic	TSC2	/	28+6	33	Positive	Positive	Negative	Lost to follow-up
16	Genetic	TSC2	/	22+6	29	Negative	Positive	Negative	Pregnancy termination
17	Clinical	/	/	28	25	Positive	Positive	Negative	Pregnancy termination
18	Genetic	TSC2	/	31+6	32	Positive	Positive	Negative	Pregnancy termination
19	Genetic	TSC2	Negative	23+3	35	Negative	Positive	Negative	Pregnancy termination
20	Genetic	TSC2	Mother	27+5	34	Negative	Positive	Negative	Term birth
21#	Genetic	TSC2	Negative	32+2	31	Positive	Positive	Negative	Fetal reduction
22	Genetic	TSC2	Negative	21+5	32	Negative	Positive	Negative	Pregnancy termination
23	Genetic	TSC2	Negative	25+5	28	Negative	Positive	Negative	Pregnancy termination
24	Genetic	TSC2	Negative	24+5	32	Positive	Positive	Negative	Pregnancy termination

TSC, Tuberous sclerosis complex; GA, Gestational age; # Twins; *MRI localizer images revealed brain lesions.

Seventeen fetuses were diagnosed with TSC via prenatal whole-exome sequencing of amniotic fluid samples, revealing one TSC1 gene variant and 16 TSC2 gene variants. Parental testing was performed in 11 families, identifying TSC-associated variants in 3 fathers and 2 mothers. One particularly noteworthy case involved no abnormalities detected by whole-exome sequencing of the father’s peripheral blood; however, targeted high-throughput sequencing of his semen revealed a mosaic variant of the *TSC2* gene (c.2713C>T). The remaining seven fetuses were diagnosed based on imaging findings consistent with the 2012 International TSC Consensus Conference diagnostic criteria.

Follow-up data were available for all cases. Outcomes included 16 pregnancy terminations, 2 selective reductions in twin pregnancies, 4 cases lost to follow-up, and 2 live births. The two live-born infants were confirmed to have TSC2 variants on postnatal whole-exome sequencing. Cardiac rhabdomyomas in both cases were monitored by ultrasound. At follow-up—6 months and 2 years 8 months after birth, respectively—both children continued to have persistent cardiac rhabdomyomas but exhibited normal growth and neurodevelopment.

### Fetal brain imaging findings

**Figure 2:**
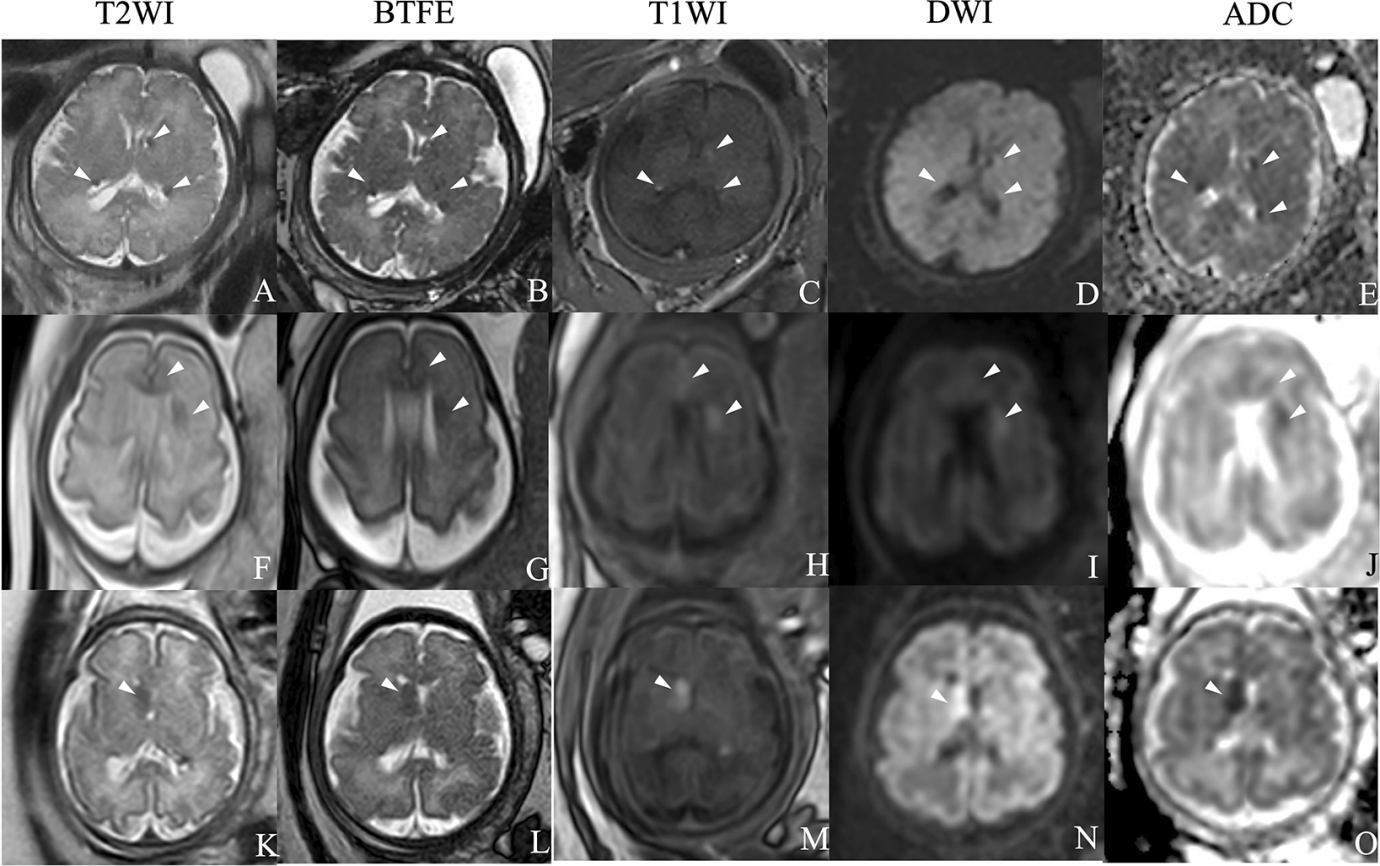
TSC-related MRI characteristics on fetal brain MRI. Cortical dysplasia appear as hyperintense signals on T1WI against the dark background of the white matter, a phenomenon often referred to as the "bright spot" sign. The white arrow represents the lesion. **(A–E)** Subependymal nodules; **(F–J)** cortical dysplasia (includes cortical tubers and white matter migrational abnormalities); **(K–O)** subependymal giant cell astrocytoma.

(**Figures 2A–O**) illustrates representative MRI features of brain lesions in fetuses with TSC. Of the 23 fetuses who underwent brain MRI, 20 were referred following prenatal ultrasound detection of cardiac masses suggestive of rhabdomyomas to evaluate for TSC. One case was referred due to a prenatal ultrasound finding suggestive of a Blake’s pouch cyst, one due to intracranial hyperechoic nodules, and another after incidental discovery of cerebral nodules during a fetal kidney MRI examination.

Of the 23 fetal brain MRI studies, 8 showed no significant abnormalities (all those 8 fetuses had cardiac rhabdomyoma and were genetically confirmed TSC). The remaining 15 fetuses exhibited intracranial lesions—14 with multiple intracranial lesions and 1 with a single lesion. Bilateral subependymal involvement was identified in 13 cases and unilateral involvement in 1 case (**Table 2**). Cortical dysplasia (including cortical nodules and radial migration lines within the white matter) was observed in 8 cases. One case of subependymal giant cell astrocytoma was identified. Lesion sizes ranged from 2.0 to 14.3 mm. Subependymal nodules appeared as nodular structures along the ventricular walls (**Figures 2A-E**), predominantly hyperintense on T1WI (12/14, 85.7%) and isointense in a minority (2/14, 14.3%). On T2WI and BTFE sequences, they appeared hypointense in all cases (100%). On DWI, most showed hyperintense signals (11/14, 78.6%). The nodules generally had well-defined margins on T2WI than on BTFE (85.7% vs. 50.0%). Cortical dysplasia included cortical tubers and white matter migrational abnormalities. Cortical dysplasia manifests as uniform hyperintensity on T1WI (8/8, 100%), mostly as hypointense on T2WI (6/8, 75%) and BTFE (5/8, 62.5%), and predominantly as hypointense on DWI (6/8, 75.0%) (**Figures 2F-J**). Cortical dysplasia appeared as hyperintense signals on T1WI against the dark background of white matter, a phenomenon often called the “bright spot” sign. Our study found that cortical dysplasia are more easily detected on T1WI than on T2WI (**Figure 3**). The boundary clarity evaluation of cortical dysplasia across various sequences is lower than that of subependymal nodules. Subependymal giant cell astrocytoma appeared as a hypointense signal on T2WI and a hyperintense signal on T1WI. Mild right lateral ventricular dilation was noted in two fetuses (**Figures 2K-O**).

**Table 2:** Fetal brain MRI features of 15 fetuses with TSC.

Fetal brain MRI	Subependymal nodules	Cortical dysplasias#	Subependymal giant cell astrocytoma
Characteristics	14	8	1
Number	multiple (13/14,92.9%)	multiple (7/8, 87.5%)	Single(1/1,100%)
Form	quasi-circular	quasi-circular/linear	irregular type
Size(mm)	2.0-10.6	2.0-8.9	14.3
T2WI	hypointense(14/14,100%)	hypointense (6/8,75%)	hypointense
T2WI boundary	well-defined margins (12/14,85.7%)	well-defined margins(3/6,50%)	well-defined margins
T1WI	hyperintense(12/14,85.7%) isointense(2/14,14.3%)	hyperintense(8/8,100%)	hyperintense
T1WI boundary	well-defined margins(11/14,78.6%)	well-defined margins(3/8,37.5%)	well-defined margins
BTFE	hypointense(14/14,100%)	hypointense(5/8,62.5%)	hypointense
BTFE boundary	well-defined margins (7/14,50.0%)	well-defined margins(2/5,40%)	well-defined margins
DWI	hyperintense(11/14,78.6%) partial high signal(1/7.1%) isointense (2/14,14.3%)	hyperintense(6/8,75%) isointense(2/8,25%)	hyperintense
ADC	hypointense(13/14,92.9%) partial low signal(1/14,7.1%)	hypointense(6/8,75%) isointense(2/8,25%)	hypointense

TSC, Tuberous sclerosis complex; T2WI, T2-weighted imaging; T1WI, T1-weighted imaging; BTFE, balanced turbo field echo; DWI, diffusion-weighted imaging; ADC, apparent diffusion coefficient.

# Cortical dysplasia includes cortical tubers and white matter migrational abnormalities.

**Figure 3:**
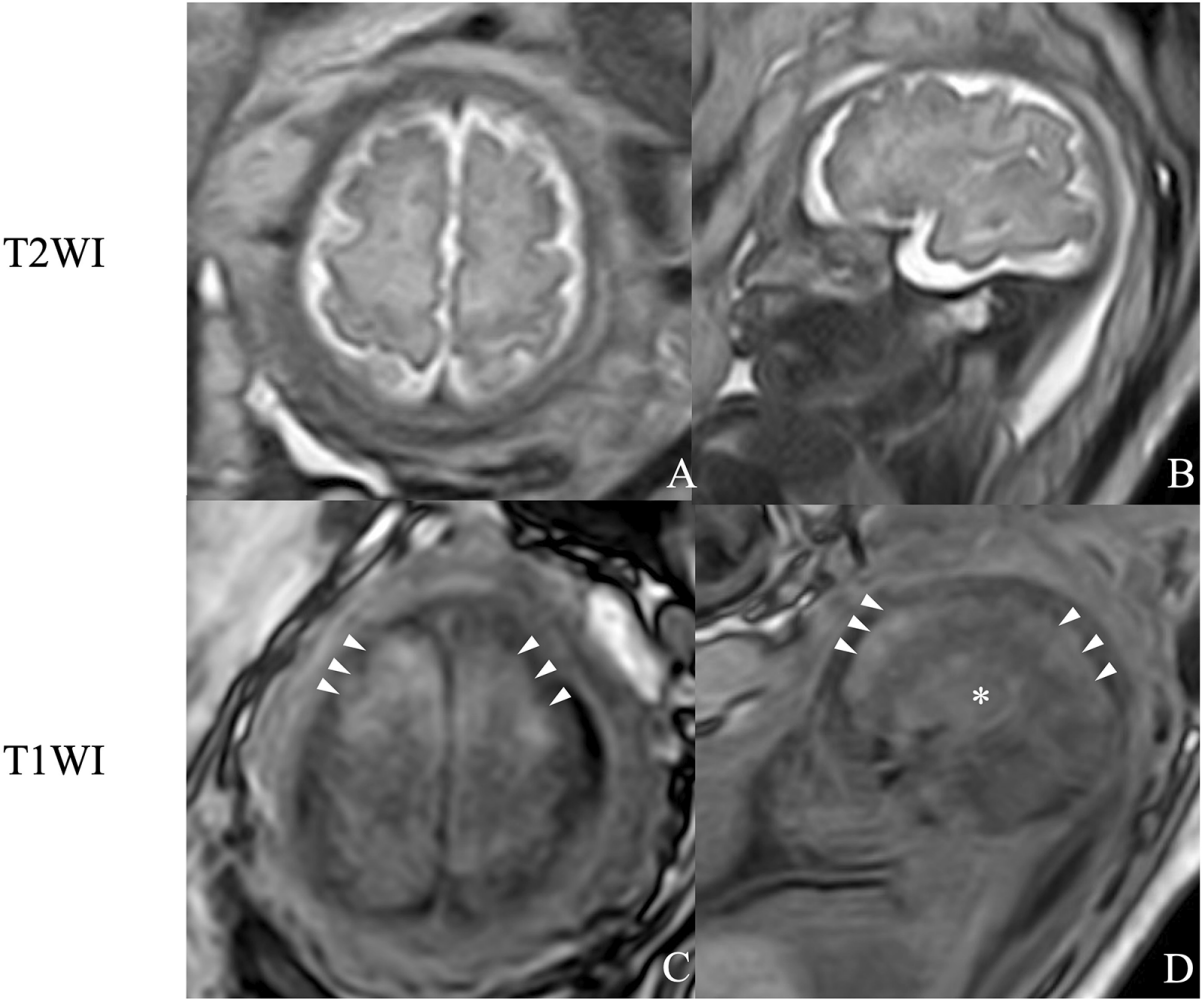
Cortical dysplasia MRI characteristics on fetal brain MRI, Cortical dysplasia is more easily detected on T1WI than on T2WI. **(A, B)** T2WI shows suspicious lesions in the fetal cortex and white matter; **(C, D)** T1WI reveals extensive cortical dysplasia in the fetus. The white arrow represents the cortical dysplasia lesion. The asterisks indicate numerous lesions also present in the basal ganglia region.

### Fetal cardiac imaging findings

**Figure 4:**
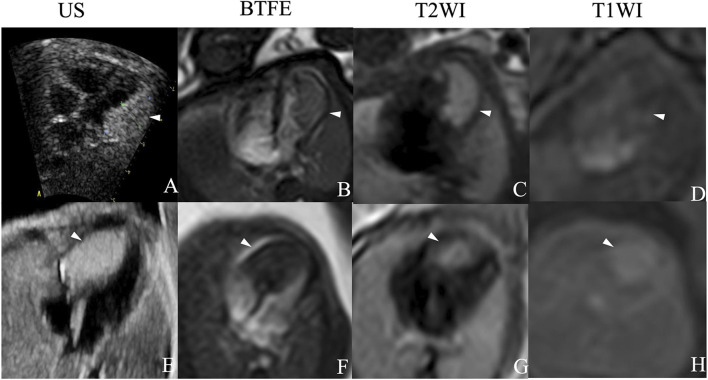
Fetal cardiac rhabdomyoma: Ultrasound and MRI findings. Cardiac rhabdomyomas appear as hyperintense signals on T2WI against the dark background of the myocardium, a phenomenon often referred to as the "bright spot" sign. The white arrow represents the lesion. **(A–D)** Rhabdomyoma in the left ventricular wall; **(E–H)** rhabdomyoma in the interventricular septum.

Fetal cardiac MRI was performed on 12 fetuses. **Table 3** and (**Figure 4A–H**) show representative MRI characteristics of cardiac rhabdomyoma. Prenatal cardiac ultrasound identified multiple lesions in 9 cases and single lesions in 3 cases. The lesions were distributed across the ventricular septum (7 cases), left ventricle (11 cases), right ventricle (5 cases), and right atrium (1 case). Cardiac MRI detected multiple lesions in 5 fetuses and single lesions in another 7. The lesion distribution was as follows: ventricular septum (7 cases), left ventricle (8 cases), right ventricle (6 cases), and right atrium (1 case). Lesions demonstrated BTFE hypointensity in all cases (100%), T1WI isointensity/hyperintensity (91.67% and 8.33%, respectively), T2WI hyperintensity and DWI hypointensity in all cases (100%). Cardiac rhabdomyomas appeared as hyperintense signals on T2WI against the dark background of the myocardium, a finding commonly known as the “bright spot” sign. Among the 12 fetuses who did not undergo cardiac MRI, 9 exhibited cardiac lesions visible on brain MRI localizer images.

**Table 3:** Fetal cardiac rhabdomyoma manifestations on ultrasound and MRI.

No	US CR number	MRI CR number	US CR location	MRI CR location	US max(mm)	MRI max(mm)	T1WI	T2WI	BTFE	DWI
1	multiple	single	IVS,LV	IVS	11x8	10.9x7.3	ISO	HYPER	HYPO	HYPO
2	single	single	RV	RV	9x8	10.7x7.6	ISO	HYPER	HYPO	HYPO
3	single	single	LV	LV	19x9	18x11	ISO	HYPER	HYPO	/
4	multiple	multiple	IVS,LV	IVS,LV,RV	8.4x6.5	8.4x4.6	ISO	HYPER	HYPO	HYPO
5	multiple	single	IVS,LV	IVS	10.9x8.9	7.0x4.7	ISO	HYPER	HYPO	HYPO
6	multiple	multiple	LV,RV,RA	LV,RV,RA	20x14	10.9x10.8	ISO	HYPER	HYPO	HYPO
7	multiple	multiple	IVS,LV,RV	IVS,LV,RV	6.7x5.5	4.8x4.5	ISO	HYPER	HYPO	HYPO
8	multiple	multiple	LV,RV	IVS,LV,RV	15x14	14x11.5	m-HYPER	HYPER	HYPO	HYPO
9	single	single	LV	LV	4.1x3.3	3.8x2.2	ISO	HYPER	HYPO	HYPO
10	multiple	multiple	IVS,LV,RV	IVS,LV,RV	10.0x6.8	7.2x4.9	ISO	HYPER	HYPO	HYPO
11	multiple	single	IVS,LV	LV	5.4x4.2	2.8x2.5	ISO	HYPER	HYPO	HYPO
12	multiple	single	IVS,LV	IVS	8x4	5.6x8.8	ISO	HYPER	HYPO	HYPO

US, ultrasound; CR, cardiac rhabdomyoma; IVS, Ventricular septum; LV, Left ventricle; RV, Right ventricle; RA, Right atrium; HYPO, Hypointense; m-HYPER, Mildly hyperintense; ISO, isointense; MRI, magnetic resonance imaging; T1WI, T1-weighted imaging; T2WI, T2-weighted imaging; BTFE, balanced turbo field echo; DWI, diffusion-weighted imaging.

### Subgroup analysis by diagnostic modality

Among the 24 fetuses included in this study, 29.2% (7/24) were diagnosed based on imaging findings, with a gestational age at diagnosis of 30.49 ± 4.58 weeks, while 70.8% (17/24) were diagnosed based on genetic testing, with a gestational age at diagnosis of 27.77 ± 4.75 weeks. In the imaging diagnosis group, all 7 cases showed abnormalities on both fetal echocardiography and brain MRI. Among them, one fetus had concomitant renal cysts, and multiple bilateral renal cysts were identified in the mothers of two cases. Regarding fetal brain MRI findings, 57.1% (4/7) of cases showed subependymal nodules, while 42.9% (3/7) showed both subependymal nodules and cortical dysplasia. In the genetic diagnosis group, fetal echocardiography revealed abnormalities in 88.2%(15/17) of cases. Among these, two cases had both fetal cardiac and brain lesions, one case presented with bilateral renal cysts, and one case showed only brain lesions. Overall, fetal brain MRI abnormalities were detected in 9 cases, accounting for 52.9% (9/17) of this group. Among the cases with abnormal fetal brain MRI findings, 33.3% (3/9) showed subependymal nodules, 55.6% (5/9) showed both subependymal nodules and cortical dysplasia, and 11.1% (1/9) showed cortical dysplasia alone.

Statistical analysis showed no correlation between the number of cardiac lesions and brain lesions (*p* = 0.23). Furthermore, genetic findings were not associated with the number of cardiac and brain lesions (both *p* > 0.05). There was no significant difference in the detection of cardiac lesions between fetuses older than 26 weeks and those younger than 26 weeks gestational age (*p* = 0.85). However, fetuses older than 26 weeks had a higher detection rate of brain lesions on MRI than those younger than 26 weeks (*p* = 0.02). Notably, 75% of cases with cortical tubers and white matter migrational abnormalities occurred in fetuses beyond 26 weeks gestation.

## Discussion

TSC is an autosomal dominant neurocutaneous disorder that can affect multiple organ systems (1). Approximately 85%–90% of cases are caused by pathogenic variants in the TSC1 and/or TSC2 genes, with considerable clinical heterogeneity (4, 13). TSC1 (hamartin) and TSC2 (tuberin) are tumor suppressor genes regulating the mTOR signaling pathway (14). Phenotypic presentation ranges from mild skin lesions to severe neurological, renal, and cardiac complications. In 10%–15% of confirmed TSC cases, no pathogenic variants are detected (2), and affected individuals typically exhibit milder symptoms.

The 2012 International Tuberous Sclerosis Complex Consensus Conference revised the 1998 diagnostic criteria and established updated guidelines (4). Based on the sensitivity and specificity of systemic manifestations, clinical diagnostic requires either (1) at least two major features or (2) one major feature and two or more minor features. Furthermore, molecular diagnosis was introduced as an independent diagnostic pathway—identification of a pathogenic *TSC1* or *TSC2* variant is sufficient for a definitive diagnosis, even in the absence of clinical features. However, given technological limitations, approximately 10%–15% of patients test negative on genetic screening (2). Thus, combining genetic testing with imaging is essential, particularly when genetic results are inconclusive. Prenatal imaging remains critical for TSC diagnosis.

Since its introduction in the 1980s, fetal ultrasound has become a cornerstone of prenatal screening and diagnosis due to its simplicity, real-time imaging capability, noninvasiveness, safety, low cost, and repeatability (15). However, fetal cardiac rhabdomyomas often represent the only detectable manifestation (16). Studies have shown that cardiac rhabdomyomas are frequently the initial feature of fetal TSC, consistent with our findings (8). In this study, 21 of 24 fetuses were referred for prenatal diagnostic evaluation after ultrasound detection of cardiac masses. Although ultrasound is highly effective for identifying fetal cardiac rhabdomyomas, its ability to detect CNS abnormalities is limited (17). MRI provides superior soft-tissue contrast, clearer visualization and delineation, and histopathological correlation, making it the preferred modality for detecting and differentiating CNS lesions (18). Fetal brain MRI can provide more comprehensive information than ultrasound (19, 20). In this study, intracranial lesions in TSC fetuses primarily manifested as bilateral subependymal nodules—findings consistent with previous reports (21).

Subependymal nodules typically appeared hyperintense on T1WI (85.7%) and hypointense on T2WI (100%), with partial hyperintensity on DWI (78.6%), consistent with previous studies (22, 23). Unlike the variable MRI signals of subependymal nodules observed after birth, these signals tend to be more consistent during the fetal period because the lesions appear more distinct against the background of unmyelinated white matter (24). Meanwhile, subependymal nodules are better visualized on T2WI than on T1WI, because the hyperintense cerebrospinal fluid in the lateral ventricles provides contrast against the hypointense nodules. Signal variation on DWI likely reflects differences in hamartoma composition and calcification levels, which influence magnetic resonance signal characteristics. Cortical dysplasia included cortical tubers and white matter migrational abnormalities. White matter migrational abnormalities, also known as radial migration lines, refer to linear or curvilinear abnormal signals extending from the periventricular region to the subcortical areas, representing aberrant migration of dysplastic stem cells along glial pathways (21). White matter migrational abnormalities have been rarely reported in previous fetal studies. Prior research indicates that cortical tubers are typically visible only on postnatal MRI, with their number and location closely linked to epilepsy risk and neurodevelopmental outcomes in children with TSC (25). However, in our study, eight fetuses showed cortical tubers and white matter migrational abnormalities, which may be related to the gestational age at the time of imaging. The presence of a “bright spot” sign on T1WI aids in diagnosis. In our study, T1WI demonstrated cortical dysplasia better than T2WI. A possible reason is that the lesions appear hyperintense on T1WI, making them easier to identify against the hypointense background. Unlike subependymal nodules, cortical dysplasia lacks the contrast provided by the hyperintense cerebrospinal fluid in the lateral ventricles on T2WI. Although MRI can detect subependymal nodules, cortical tubers, and white matter migrational abnormalities, some lesions may develop later, highlighting the need for follow-up imaging during the third trimester to avoid missed diagnoses. We found that the detection rate of fetal intracranial lesions was higher after 26 weeks of gestation. The higher detection rate of intracranial lesions after 26 weeks of gestation may reflect lesion progression or may be attributable to improved lesion detectability later in gestation. Goergen and Fahey found that TSC fetuses with abnormal brain MRI findings were examined at a later gestational age than those with negative MRI findings (12). In their study, among 42 TSC fetuses with abnormal MRI findings, only 9 were identified at ≤24 weeks’ gestation. This is broadly consistent with our findings. As this was a retrospective study and most fetuses did not undergo serial MRI examinations, we were unable to reliably distinguish biological progression from improved imaging detectability. Future prospective longitudinal studies incorporating serial fetal MRI are warranted to clarify the temporal evolution and detectability of intracranial lesions in fetal TSC.

For our case 5, fetal brain MRI revealed multiple punctate and nodular lesions in the subependymal regions of both ventricles. Whole-exome sequencing identified a *TSC2* gene variant, and cytomegalovirus (CMV) nucleic acid was detected in amniotic fluid. Therefore, intracranial nodules should be differentiated among TSC, CMV infection, and neuronal migration disorders. In fetuses, TSC lesions typically appear hyperintense on T1WI and hypointense on T2WI. CMV infection can cause intracranial calcifications, microcephaly, cerebral atrophy, and ventriculomegaly. Heterotopic gray matter nodules demonstrate the same signal intensity as cortical gray matter across all MRI sequences.

Fetal cardiac MRI revealed that cardiac rhabdomyomas were mainly located in the left ventricle, interventricular septum, and right ventricle, consistent with literature reports (26). Atrial rhabdomyomas are rarely observed; only one right atrial case was identified in our cohort. Advances in rapid imaging have made fetal cardiac MRI feasible, showing good capability for detecting cardiac masses (12). However, discrepancies between MRI and ultrasound findings likely result from the absence of ECG-gating in cardiac MRI, which can introduce motion artifacts. Studies show that ECG-gating enhances image quality and enables functional and hemodynamic cardiac assessments (27, 28). MRI signal characteristics can aid in differential diagnosis: cardiac rhabdomyomas appear as hyperintense signals on T2WI against the dark background of the myocardium, a phenomenon often referred to as the “bright spot” sign 0. Cardiac rhabdomyomas are often multiple. Emerging evidence indicates that TSC2 mutations, compared with TSC1, are more often associated with severe cardiac dysfunction and adverse outcomes. Prior research has indicated that mechanically obstructive cardiac rhabdomyomas can lead to intrauterine fetal death (29). hemodynamic Fetal cardiac rhabdomyomas may undergo dynamic changes during both the prenatal and postnatal periods. D’Addario et al. demonstrated that two-dimensional ultrasonography and Doppler ultrasonography are effective noninvasive methods for diagnosing fetal cardiac rhabdomyomas and monitoring their impact on fetal cardiac function (30). Bader et al. reported that most fetuses with cardiac rhabdomyomas were subsequently diagnosed with TSC, and that some neonates developed cardiac symptoms requiring medical treatment or surgical intervention (31). Kavgacı and Arı reported four cases of fetal cardiac rhabdomyoma diagnosed by echocardiography and showed that these tumors may exhibit growth, stability, or regression (32). These findings highlight the importance of early prenatal recognition, genetic counseling, and individualized multidisciplinary follow-up after birth. Okutucu et al. also reported that the median gestational age at diagnosis of fetal cardiac rhabdomyoma was 26 weeks, and that the left ventricle was the most commonly involved site (33). Peng et al. retrospectively analyzed 54 fetuses with cardiac rhabdomyomas and found that multiple lesions were common (34). Among live-born infants with follow-up, most cardiac lesions decreased in size or disappeared, whereas some remained stable. These findings are consistent with the natural history of cardiac rhabdomyomas. Fetal echocardiography remains the primary imaging modality for the detection and evaluation of fetal cardiac rhabdomyomas. In addition, the combination of fetal echocardiography and cardiac MRI, including the assessment of ejection fraction, hemodynamics, and tissue characteristics, may provide a more comprehensive evaluation of affected fetuses.

Currently, no curative therapy exists for TSC; management focuses on symptomatic control. Consistent with prior studies, heterozygous *TSC2* variants were more prevalent than *TSC1* variants, accounting for 94.1% of cases in this study (35). Most TSC cases result from *de novo* mutations, making fetal risk prediction from parental testing challenging. Among the 17 fetuses who underwent amniocentesis, parental testing was performed in 11 families. Results demonstrated marked genetic heterogeneity: six parents showed no abnormalities, while five carried pathogenic variants—one father with a *TSC1* mutation, two mothers and one father with *TSC2* mutations, and one father with a mosaic *TSC2* variant detected only in semen. No abnormalities were found in his peripheral blood whole-exome sequencing. Recent research findings suggest that when no exonic TSC1 and TSC2 mutations are identified, intronic splice-site or somatic mosaic variants may be responsible. Therefore, next-generation sequencing covering the full *TSC1* and *TSC2* gene sequences is recommended for comprehensive testing (1, 36). Early identification of genetic variants and mosaicism can improve risk prediction and clinical management in infants and children with TSC. As 10%–15% of patients may test negative on genetic screening, prenatal imaging plays a vital role in improving diagnostic yield (14). Furthermore, assessing the risk of epilepsy and determining the need for early EEG monitoring are essential. Approximately 80%–90% of patients with TSC develop epilepsy, and approximately 80% of these cases become drug-resistant. Early detection of cortical tubers and white matter migrational abnormalities is crucial for preventive management. With improved understanding of TSC pathogenesis, mTOR inhibitors—targeted agents addressing the underlying molecular defect—have been introduced into clinical practice to control disease progression.

Isolated cardiac rhabdomyomas without other lesions often indicate a better prognosis (37, 38). In this study, both fetuses with rhabdomyomas who were followed had favorable outcomes. The rate of pregnancy termination was relatively high, reflecting the independent decisions of families. There is an urgent need to improve prenatal counseling to help parents make better-informed choices, including offering imaging evaluations for parents with *TSC* gene mutations. Notably, one mother in this study was found to have cortical tubers on her brain MRI despite showing no clinical symptoms, which influenced her decision to continue the pregnancy. Furthermore, early use of mTOR inhibitors may improve clinical symptoms and modify both clinical and imaging findings. Recent case reports have documented successful treatment with mTOR inhibitors during the fetal period (39), suggesting a promising early intervention strategy for fetuses with TSC.

The limitations of this study are as follows. First, this study had a retrospective design and a relatively small sample size. In addition, postnatal imaging and long-term clinical follow-up were not available for all cases. Consequently, the associations between prenatal MRI findings and postnatal epilepsy, neurodevelopmental outcomes, or other clinical manifestations could not be comprehensively evaluated. Future studies will include larger cohorts, standardized postnatal imaging, and long-term clinical follow-up. Second, most cases were referred because of cardiac rhabdomyomas suspected on prenatal ultrasound, which may have introduced referral bias. Therefore, our MRI phenotype description may not be fully generalizable to the broader prenatal TSC population, particularly to cases identified through routine brain screening rather than cardiac findings. Finally, fetal cardiac MRI was performed without cardiac gating, precluding the assessment of cardiac function and hemodynamics. In future cases, Doppler ultrasound gating will be used to acquire cardiac cine images.

## Conclusion

This study demonstrates that prenatal MRI enables accurate diagnosis of TSC, highlighting that the “bright spot” sign is an important feature of fetal TSC on MRI. Prenatal ultrasound remains the primary imaging modality for diagnosing cardiac rhabdomyomas. Integrating ultrasound with MRI enhances both the diagnostic accuracy for cardiac rhabdomyomas and the detection rate of TSC-related intracranial lesions. When combined with genetic testing for TSC, this multimodal diagnostic strategy enables more comprehensive prenatal evaluation, counseling, and prognostic assessment for affected families.

## Data Availability

The raw data supporting the conclusions of this article will be made available by the authors, without undue reservation.

## References

[1] Henske EP Jóźwiak S Kingswood JC Sampson JR Thiele EA . Tuberous sclerosis complex. Nat Rev Dis Primers. (2016) 2:16035. doi: 10.1038/nrdp.2016.35 27226234

[2] Curatolo P Bombardieri R Jozwiak S . Tuberous sclerosis. Lancet. (2008) 372:657–68. doi: 10.1016/S0140-6736(08)61279-9 18722871

[3] Randle SC . Tuberous sclerosis complex: a review. Pediatr Ann. (2017) 46:e166–71. doi: 10.3928/19382359-20170320-01 28414398

[4] Northrup H Krueger DA International Tuberous Sclerosis Complex Consensus Group . Tuberous sclerosis complex diagnostic criteria update: recommendations of the 2012 international tuberous sclerosis complex consensus conference. Pediatr Neurol. (2013) 49:243–54. doi: 10.1016/j.pediatrneurol.2013.08.001 24053982 PMC4080684

[5] DiMario FJ Sahin M Ebrahimi-Fakhari D . Tuberous sclerosis complex. Pediatr Clin North Am. (2015) 62:633–48. doi: 10.1016/j.pcl.2015.03.005 26022167

[6] Ebrahimi-Fakhari D Mann LL Poryo M Graf N von Kries R Heinrich B . Incidence of tuberous sclerosis and age at first diagnosis: new data and emerging trends from a national, prospective surveillance study. Orphanet J Rare Dis. (2018) 13:117. doi: 10.1186/s13023-018-0870-y 30016967 PMC6050673

[7] Dragoumi P O'Callaghan F Zafeiriou DI . Diagnosis of tuberous sclerosis complex in the fetus. Eur J Paediatr Neurol. (2018) 22:1027–34. doi: 10.1016/j.ejpn.2018.08.005 30279084

[8] Milon V Malinge MC Blanluet M Tessarech M Battault C Prestwich S . Diagnosis of tuberous sclerosis in the prenatal period: a retrospective study of 240 cases and review of the literature. Eur J Hum Genet. (2024) 32:1590–8. doi: 10.1038/s41431-024-01631-w 38806662 PMC11606953

[9] Aertsen M Diogo MC Dymarkowski S Deprest J Prayer D . Fetal MRI for dummies: what the fetal medicine specialist should know about acquisitions and sequences. Prenat Diagn. (2020) 40:6–17. doi: 10.1002/pd.5579 31618472

[10] Xie L Xu H He X Fu H Zhang L Bai W . The potential of 1.5 T magnetic resonance imaging for the evaluation of fetal anomalies of the great vessels. Front Pediatr. (2023) 11:1136892. doi: 10.3389/fped.2023.1136892 37056942 PMC10086421

[11] Bekiesinska-Figatowska M Sobieraj P Pasieczna M Szymkiewicz-Dangel J . Early diagnosis of tuberous sclerosis complex: prenatal diagnosis. AJNR Am J Neuroradiol. (2023) 44:1070–6. doi: 10.3174/ajnr.A7952 37536734 PMC10494953

[12] Goergen SK Fahey MC . Prenatal MR imaging phenotype of fetuses with tuberous sclerosis: an institutional case series and literature review. AJNR Am J Neuroradiol. (2022) 43:633–8. doi: 10.3174/ajnr.A7455 35332020 PMC8993194

[13] Dabora SL Jozwiak S Franz DN Roberts PS Nieto A Chung J . Mutational analysis in a cohort of 224 tuberous sclerosis patients indicates increased severity of TSC2, compared with TSC1, disease in multiple organs. Am J Hum Genet. (2001) 68:64–80. doi: 10.1086/316951 11112665 PMC1234935

[14] Jozwiak J Wlodarski P . Hamartin and tuberin modulate gene transcription via beta-catenin. J Neuro-Oncol. (2006) 79:229–34. doi: 10.1007/s11060-006-9134-0 16552619

[15] Karim JN Campbell H Pandya P Wilson ECF Alfirevic Z Chudleigh T . Clinical and cost-effectiveness of detailed anomaly ultrasound screening in the first trimester: a mixed-methods study. Health Technol Assess. (2025) 29:1–250. doi: 10.3310/NLTP7102 40455571 PMC12146947

[16] Giacoia GP . Fetal rhabdomyoma: a prenatal echocardiographic marker of tuberous sclerosis. Am J Perinatol. (1992) 9:111–4. doi: 10.1055/s-2007-994681 1590864

[17] Calixto C Taymourtash A Karimi D Snoussi H Velasco-Annis C Jaimes C . Advances in fetal brain imaging. Magn Reson Imaging Clin N Am. (2024) 32:459–78. doi: 10.1016/j.mric.2024.03.004 38944434 PMC11216711

[18] Cortes-Albornoz MC Bedoya MA Choi JJ Jaimes C . MR insights into fetal brain development: what is normal and what is not. Pediatr Radiol. (2024) 54:635–45. doi: 10.1007/s00247-024-05890-z 38416183 PMC13157681

[19] Goergen S Furruqh F Evans R Cicilet S Mankad K . Algorithmic approach to neuroradiological diagnosis with pre-natal MRI: non-visualization of the fetal cavum septi pellucidi on mid-trimester screening ultrasound. Br J Radiol. (2023) 96:20221042. doi: 10.1259/bjr.20221042 36930694 PMC10321250

[20] Fileva N Severino M Tortora D Ramaglia A Paladini D Rossi A . Second trimester fetal MRI of the brain: through the ground glass. J Clin Ultrasound. (2023) 51:283–99. doi: 10.1002/jcu.23423 36785503

[21] Rovira À Ruiz-Falcó ML García-Esparza E López-Laso E Macaya A Málaga I . Recommendations for the radiological diagnosis and follow-up of neuropathological abnormalities associated with tuberous sclerosis complex. J Neuro-Oncol. (2014) 118:205–23. doi: 10.1007/s11060-014-1429-y 24771286

[22] Adamsbaum C Merzoug V Kalifa G . Imaging of CNS manifestations of tuberous sclerosis in children. J Neuroradiol. (2005) 32:204–9. doi: 10.1016/s0150-9861(05)83138-4 16134302

[23] Gelot AB Represa A . Progression of fetal brain lesions in tuberous sclerosis complex. Front Neurosci. (2020) 14:899. doi: 10.3389/fnins.2020.00899 32973442 PMC7472962

[24] Baron Y Barkovich AJ . MR imaging of tuberous sclerosis in neonates and young infants. AJNR Am J Neuroradiol. (1999) 20:907–16. PMC7056154 10369365

[25] Goel R Aggarwal N Lemmon ME Bosemani T . Fetal and maternal manifestations of tuberous sclerosis complex: value of fetal MRI. Neuroradiol J. (2016) 29:57–60. doi: 10.1177/1971400915621323 26838171 PMC4978341

[26] Pipitone S Mongiovì M Grillo R Gagliano S Sperandeo V . Cardiac rhabdomyoma in intrauterine life: clinical features and natural history. A case series and review of published reports. Ital Heart J. (2002) 3:48–52. 11899590

[27] Suchá D Bohte AE van Ooij P Leiner T Schrauben EM Grotenhuis HB . Fetal cardiovascular magnetic resonance: history, current status, and future directions. J Magn Reson Imaging. (2025) 61:2357–75. doi: 10.1002/jmri.29664 39578988 PMC12063768

[28] Vollbrecht TM Bissell MM Kording F Geipel A Isaak A Strizek BS . Fetal cardiac MRI using Doppler US gating: emerging technology and clinical implications. Radiol Cardiothorac Imaging. (2024) 6:e230182. doi: 10.1148/ryct.230182 38602469 PMC11056758

[29] Holley DG Martin GR Brenner JI Fyfe DA Huhta JC Kleinman CS . Diagnosis and management of fetal cardiac tumors: a multicenter experience and review of published reports. J Am Coll Cardiol. (1995) 26:516–20. doi: 10.1016/0735-1097(95)80031-b 7608458

[30] D'Addario V Pinto V Di Naro E Del Bianco A Di Cagno L Volpe P . Prenatal diagnosis and postnatal outcome of cardiac rhabdomyomas. J Perinat Med. (2002) 30:170–5. doi: 10.1515/JPM.2002.022 12012639

[31] Bader RS Chitayat D Kelly E Ryan G Smallhorn JF Toi A . Fetal rhabdomyoma: prenatal diagnosis, clinical outcome, and incidence of associated tuberous sclerosis complex. J Pediatr. (2003) 143:620–4. doi: 10.1067/S0022-3476(03)00494-3 14615733

[32] Kavgacı A Arı ME . Dynamic evolution of fetal cardiac rhabdomyomas: prenatal diagnosis and postnatal echocardiographic insights. Eur J Pediatr. (2025) 184:332. doi: 10.1007/s00431-025-06164-y 40343543

[33] Okutucu G Tanacan A Sahin D . Clinical outcomes of fetuses with cardiac rhabdomyoma: a case series from a tertiary center. J Obstetrics Gynaecology Res. (2024) 50:342–50. doi: 10.1111/jog.15846 38062975

[34] Peng L Cai Y Wu J Ling W Wu Q Guo S . Prenatal diagnosis and clinical management of cardiac rhabdomyoma: a single-center study. Front Cardiovasc Med. (2024) 11:1340271. doi: 10.3389/fcvm.2024.1340271 38433754 PMC10904520

[35] Chen CS Aylett CHS . New insights into tuberous sclerosis complex: from structure to pathogenesis. Front Cell Dev Biol. (2025) 13:1595867. doi: 10.3389/fcell.2025.1595867 40655946 PMC12245910

[36] Tyburczy ME Dies KA Glass J Camposano S Chekaluk Y Thorner AR . Mosaic and intronic mutations in TSC1/TSC2 explain the majority of TSC patients with no mutation identified by conventional testing. PloS Genet. (2015) 11:e1005637. doi: 10.1371/journal.pgen.1005637 26540169 PMC4634999

[37] Krueger DA Northrup H International Tuberous Sclerosis Complex Consensus Group . Tuberous sclerosis complex surveillance and management: recommendations of the 2012 International Tuberous Sclerosis Complex Consensus Conference. Pediatr Neurol. (2013) 49:255–65. doi: 10.1016/j.pediatrneurol.2013.08.002 24053983 PMC4058297

[38] Tworetzky W McElhinney DB Margossian R Moon-Grady AJ Sallee D Goldmuntz E . Association between cardiac tumors and tuberous sclerosis in the fetus and neonate. Am J Cardiol. (2003) 92:487–9. doi: 10.1016/s0002-9149(03)00677-5 12914889

[39] Cavalheiro S da Costa MDS Richtmann R . Everolimus as a possible prenatal treatment of in utero diagnosed subependymal lesions in tuberous sclerosis complex: a case report. Childs Nerv Syst. (2021) 37:3897–9. doi: 10.1007/s00381-021-05218-4 34008055

